# Reporting quality and risk of bias of randomized controlled trials of Chinese herbal medicine for multiple sclerosis

**DOI:** 10.3389/fimmu.2024.1429895

**Published:** 2024-08-19

**Authors:** Jing-Ying Wu, Jiang-Li Yang, Jia-Ling Hu, Shan Xu, Xiao-Jie Zhang, Shi-Yan Qian, Min-Li Chen, Mahad Abdulkadir Ali, Juan Zhang, Zheng Zha, Guo-Qing Zheng

**Affiliations:** ^1^ Department of Neurology, The First Affiliated Hospital of Zhejiang Chinese Medical University (Zhejiang Provincial Hospital of Traditional Chinese Medicine), Hangzhou, China; ^2^ Science and Technology Innovation Center, Guangzhou University of Chinese Medicine, Guangzhou, China

**Keywords:** autoimmune diseases, natural products, traditional Chinese medicine, herbal, quality of reporting, ROB

## Abstract

**Background:**

Multiple sclerosis (MS) is the most common non-traumatic disabling disease affecting young adults. A definitive curative treatment is currently unavailable. Many randomized controlled trials (RCTs) have reported the efficacy of Chinese herbal medicine (CHM) on MS. Because of the uncertain quality of these RCTs, the recommendations for routine use of CHM for MS remain inconclusive. The comprehensive evaluation of the quality of RCTs of CHM for MS is urgent.

**Methods:**

Nine databases, namely, PubMed, Embase, Web of Science, Cochrane Library, EBSCO, Sinomed, Wanfang Database, China National Knowledge Infrastructure, and VIP Database, were searched from inception to September 2023. RCTs comparing CHM with placebo or pharmacological interventions for MS were considered eligible. The Consolidated Standards of Reporting Trials (CONSORT) and its extension for CHM formulas (CONSORT-CHM Formulas) checklists were used to evaluate the reporting quality of RCTs. The risk of bias was assessed using the Cochrane Risk of Bias tool. The selection criteria of high-frequency herbs for MS were those with cumulative frequency over 50% among the top-ranked herbs.

**Results:**

A total of 25 RCTs were included. In the included RCTs, 33% of the CONSORT items and 21% of the CONSORT-CHM Formulas items were reported. Eligibility title, sample size calculation, allocation concealment, randomized implementation, and blinded description in CONSORT core items were reported by less than 5% of trials. For the CONSORT-CHM Formulas, the source and authentication method of each CHM ingredient was particularly poorly reported. Most studies classified the risk of bias as “unclear” due to insufficient information. The top five most frequently used herbs were, in order, *Radix Rehmanniae Preparata*, *Radix Rehmanniae Recens*, *Herba Epimedii*, *Scorpio*, and *Poria*. No serious adverse effect had been reported.

**Conclusions:**

The low reporting of CONSORT items and the unclear risk of bias indicate the inadequate quality of RCTs in terms of reporting completeness and result validity. The CONSORT-CHM Formulas appropriately consider the unique characteristics of CHM, including principles, formulas, and Chinese medicinal substances. To improve the quality of RCTs on CHM for MS, researchers should adhere more closely to CONSORT-CHM Formulas guidelines and ensure comprehensive disclosure of all study design elements.

## 1 Introduction

Multiple sclerosis (MS) is an autoimmune-mediated degenerative disease of the central nervous system (CNS), characterized by inflammatory demyelination (1). MS is the most common non-traumatic disabling disease to affect young adults (2). MS can lead to muscle weakness, sensory deficits, cognitive impairment, and fatigue (3), which ultimately affect quality of life. Despite extensive research, the underlying pathophysiology of MS remains poorly elucidated, and a definitive curative treatment is currently unavailable (4). The current treatment of MS mainly includes hormone therapies in the acute phase, disease-modifying therapies (DMTs), and symptomatic therapies in the remission phase (2). DMTs have been shown to decrease the frequencies of relapse and the accumulation of disability (5). However, the exacerbation and even new occurrence of several autoimmune diseases associated with MS have been reported as a result of DMTs (6). A network meta-analysis of the Cochrane database found that drugs used for immunotherapy may increase withdrawals (7). The American Academy of Neurology practice guideline on the efficacy and safety of DMTs in MS mentions the following (8): “Immunosuppressive medications may increase the risk of opportunistic infection and malignancy, especially with prolonged use”. Cryptococcal infections with fingolimod use and herpes family virus infections with natalizumab use have been reported (9, 10). DMTs may also cause other adverse events, including heart blocks, bradycardia, macular edema, and secondary autoimmune adverse effects (11).

Many patients with MS resorted to modalities of complementary and alternative medicine, which is used by 57%–81% of patients with MS in developed countries (12). More and more research focused on the efficacy of Chinese herbal medicine (CHM) on MS. For example, astragaloside IV (ASI) is an active monomer isolated from the Chinese medicine *Astragalus membranaceus*. In mice with experimental autoimmune encephalomyelitis (EAE), an ideal animal model for MS, early administration of ASI can delay onset and reduce disease severity (13). Bushen Yisui Formula contains 10 kinds of herbs, such as *Radix Rehmanniae Preparata, Radix Rehmanniae Recens, Scorpio*, and *Polygonum Multiflorum*, and exhibits neuroprotective effect against EAE by promoting oligodendrocyte progenitor cells’ proliferation and differentiation, thus facilitating remyelination (14). Two meta-analyses (15, 16) have proved the effectiveness of CHM in the treatment of MS. There are also a large number of randomized controlled trials (RCTs) demonstrating the effectiveness of CHM for MS. High-quality RCTs, particularly double-blind placebo-controlled trials, are generally considered to be the highest level of evidence for judging the therapeutic efficacy and safety of interventions. The credibility of the evidence supporting treatment depends on the quality of RCTs. However, an overwhelming body of evidence suggests that the quality of RCT reports remains sub-optimal (17). The reporting of methodology and bias in herbal RCTs is particularly inadequate (18). One review concluded that less than 10% of herbal medicine trials used an appropriate randomization method (19). It is noteworthy that no research has evaluated the quality of RCTs of CHM on MS currently.

The Consolidated Standards of Reporting Trials (CONSORT) checklist is commonly used to evaluate the reporting quality of RCT. Introduced in 1996 (20), it was further revised in the current 2010 version. Compliance with CONSORT has been studied in many medical fields with the general conclusion that reporting quality needs to be improved (21–23). Different CHM formulas exhibit discrepancies in composition, dosage, and duration of interventions, which may translate into high variability and low reproducibility in the outcomes assessed. Therefore, we need an adequately robust design of RCT about CHM formulas. The CONSORT extension for CHM Formulas was created in 2017 (24).

Reporting checklists just evaluate whether a study is reported in detail or not, or if important information is provided to allow reproducibility (25). They do not assess whether the procedure reported was, in fact, the correct one to use. Therefore, reporting checklists do not have adequate content validity to assess whether a study is of good/bad quality or whether a study has a high or low risk of bias (26). The Cochrane Risk of Bias tool was developed to assess the degree to which the results of a study “should be believed” (27). Therefore, we used the CONSORT, CONSORT-CHM Formulas checklists, and the Cochrane Risk of Bias tool simultaneously for assessing the quality of the RCTs of CHM for MS in terms of completeness of reporting and validity of results, to provide a more comprehensive update on RCTs that investigated the efficacy and safety of CHM in the treatment of MS.

## 2 Methods

### 2.1 Data sources and search

Nine databases, namely, PubMed, Embase, Web of Science, Cochrane Library, EBSCO, Sinomed, Wanfang Database, China National Knowledge Infrastructure, and VIP Database, were searched from the establishment of the database to September 2023. MS and its synonyms in combination with the terms of CHM or their proprietary names were used as search terms, all as MeSH and as free-text words. Chinese databases were also searched using the above search terms in Chinese.

### 2.2 Eligibility criteria

The study design was RCTs that evaluate CHM in the treatment of MS, regardless of language or publication status. Patients were diagnosed with MS according to recognized criteria internationally, such as Poser (28) and McDonald (29, 30), regardless of sex, age, race/ethnicity, geographical residence, or course of disease. The experimental group used CHM as monotherapy or adjuvant therapy, not including acupuncture and needle punching. There is no restriction on the frequency and dosage of CHM. The duration of the treatment course was at least 3 weeks. The intervention for the control group was placebo plus Western medicine (WM), or WM alone or placebo alone.

### 2.3 Exclusion criteria

Exclusion criteria included animal experiments, case reports, reviews, retrospective studies, repeated publications, and historical controlled studies.

### 2.4 Data extraction

Two independent researchers (JW and JY) extracted the following data from the included studies using a standard table, including author, year, the criteria for diagnosing MS, intervention, dosage, control, number of participants, age, sex, treatment duration, follow-up period, outcome measures, and intergroup differences. Any disagreements on data extraction were resolved by a third reviewer (SX).

### 2.5 Reporting quality

We used the CONSORT 2010 and CONSORT-CHM Formulas 2017 checklists as assessment tool for reporting quality. Two researchers (JW and JH) independently extracted information according to two checklists, who were blinded to each other’s ratings. “1” or “0” was scored to represent whether the RCT had reported the relevant item/subitem or not. “0” indicates no description of the corresponding item/subitem, and “1” indicates that the author had mentioned the description of the item/subitem in the report. Discrepancies were resolved by a third reviewer (MC). Furthermore, we summarized the CONSORT checklist into five sections: Title/Abstract and Introduction, Methods, Results, Discussion, and Other Information. We also grouped and compared manuscripts revised before CONSORT (before 2010) and after it has been revised. The CONSORT-CHM Formulas checklist was published in 2017, and we compared manuscripts published before with those published after 2017.

### 2.6 Risk of bias

The risk of bias was evaluated by using the RCT risk of bias assessment tool recommended by the Cochrane Handbook (31) and was performed independently by two researchers (JW and JH). If there was disagreement, they were discussed with and resolved by a third reviewer (SQ).

### 2.7 Description of the CHMs

The selection criteria of high-frequency herbs in the treatment of MS were those with cumulative frequencies over 50% among the top-ranked herbs. We also summarized the mechanisms of Chinese medicine monomers for MS that have been reported in previous studies.

### 2.8 Data analysis

We used Microsoft Excel 2016 for descriptive statistical analysis and counted the total number of RCTs corresponding to each CONSORT project. The subsequent results shown as percentages and 95% confidence intervals (CIs) were calculated for each overall ratio. SPSS (version 25.0) was used for statistical calculation. The significance level was presumed as *p* < 0.05.

## 3 Results

### 3.1 Study selection

**Figure 1:**
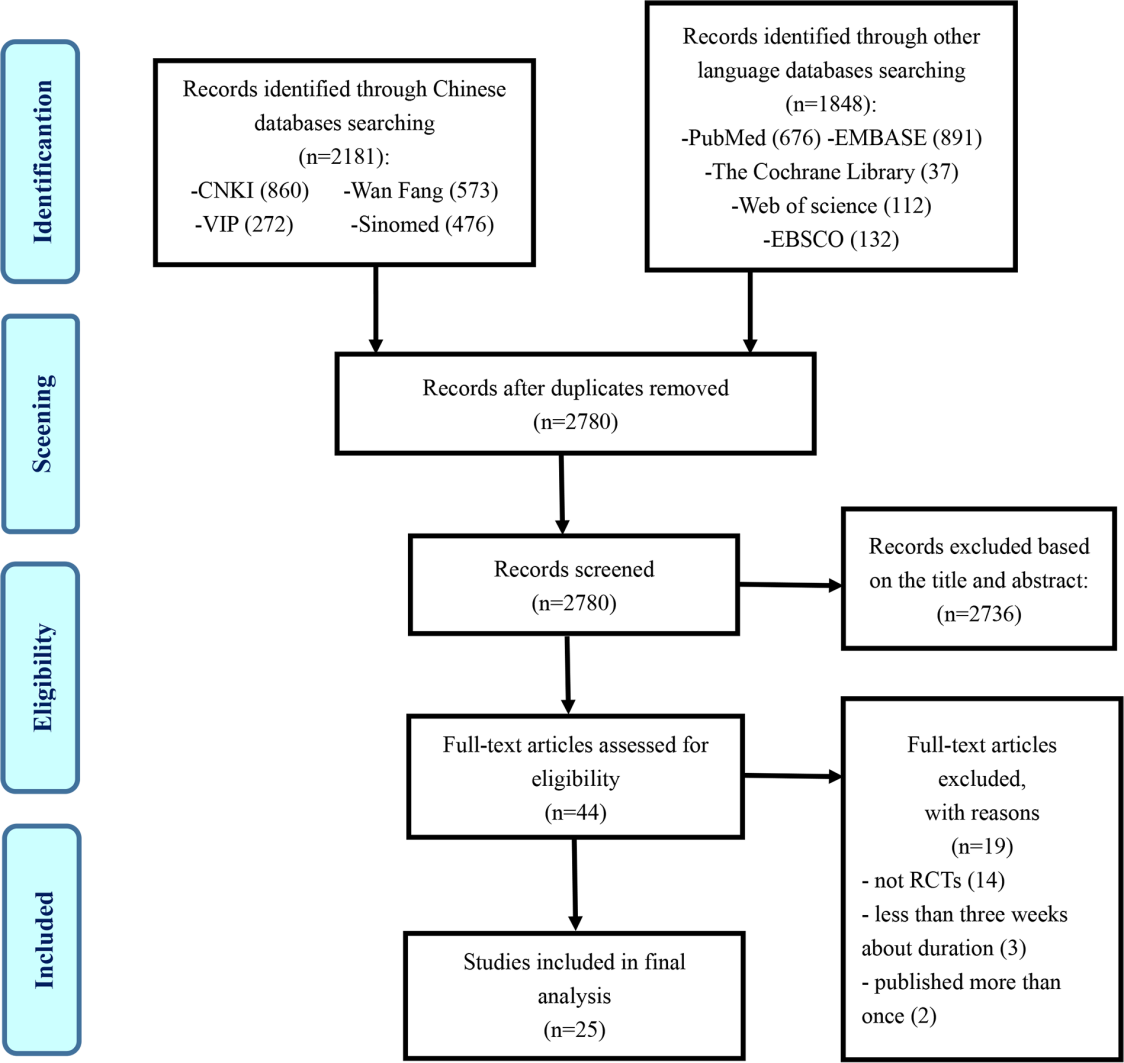
Flowchart of research selection process.

The flowchart of the research selection process is listed in **Figure 1**. A total of 4,029 potentially relevant articles were identified, and 1,249 duplicates were excluded. Another 2,736 articles were excluded through screening titles and abstracts, leaving 44 articles for further evaluation. Nineteen studies were excluded for the following reasons: 14 articles were not RCTs, 3 articles were less than 3 weeks in the duration of treatment course, and 2 articles were suspected of being published more than once. Ultimately, 25 eligible studies were selected in this study (32–56).

### 3.2 Study characteristics

**Table 1** shows partial information regarding the study characteristics. More details about the studies are shown in **Additional file 1**. A total of 1,450 participants were included in the 25 studies from 2004 to 2020, of whom 742 were in the treatment group, and 708 were in the control group. The ages ranged from 20 to 75 years old. The participants were 830 women and 607 men. Two studies (44, 45) did not report the sex of the participants. All the studies did not report sex disparity in response to treatment. All of the studies were conducted in China. One study was published in English (46), and the other 24 studies were published in Chinese. Eight studies (32, 33, 35–38, 53, 55) were diagnosed according to the Poser criteria; 17 studies (34, 39–52, 54, 56) were diagnosed according to the McDonald criteria. A total of 24 RCTs used CHM in combination with WM as the treatment group, and only 1 RCT used CHM alone as the treatment group. The duration of the studies lasted from 3 weeks to 135 days. As for outcome measure, 15 studies (32, 34–37, 40, 41, 43, 46, 48–50, 52, 54, 56) used the Expanded Disability Status Score (EDSS), 7 studies (32, 36, 37, 44, 46, 48, 49) used annual relapse frequency, 2 studies (46, 55) used annual relapse rate, and 2 studies (42, 44) used annual relapse interval. The total clinical efficacy rate was observed in 16 studies (32–35, 38–41, 43, 45, 51–56). Two studies used (50, 53) the Barthel index, and 2 studies (53, 56) used the SF-36 score.

**Table 1:** The characteristics of the included trials.

Study	Trial	Trial (male/female; age; duration)	Control (male/female; age; duration)	Treatment during	Outcome measure	Intergroup differences
Wu et al., 2020 (56)	Yangganyishen formula plus–minus 1 dose/d (200 mL) + CT	22 (M: 9, F: 13) Mean age: 36.80 y Mean disease duration: 3.00 ± 1.50 y	23 (M: 10, F: 13) Mean age: 35.40 y Mean disease duration: 4.30 ± 0.90 y	1 m	1. EDSS 2. Total clinical efficacy rate 3. SF-36 score	1. *p* < 0.05 2. *p* < 0.05 3. *p* < 0.05
Shi 2020 (55)	Improved pingfu decoction 2–3 doses/w (150–200 mL) + CT	35 (M: 18, F: 17) Mean age: 31.49 ± 2.06 y Mean disease duration: 4.01 ± 1.20 y	35 (M: 15, F: 20) Mean age: 32.22 ± 2.15 y Mean disease duration: 3.58 ± 1.21 y	1 m	1. Total clinical efficacy rate 2. Annual relapse rate	1. *p* < 0.01 2. *p* < 0.05
Qian and Wang 2020 (54)	Ziyinguben granule 6 g/tid + CT	30 (M: 12, F: 18) Mean age: 33.47 ± 11.15 y Mean disease duration: 19.38 ± 7.12 m	30 (M: 10, F: 20) Mean age: 34.07 ± 10.92 y Mean disease duration: 19.41 ± 6.11 m	27 d	1. EDSS 2. Total clinical efficacy rate 3. Neurological deficit scale	1. *p* < 0.05 2. *p* < 0.05 3. *p* < 0.05
Huang et al., 2018 (53)	Bushentianjing formula 1 dose/d (400 mL) + CT	21 (M: 9, F: 12) Mean age: 43.4 ± 10.5 y Mean disease duration: 17.3 ± 11.8 m	21 (M: 10, F: 11) Mean age: 41.9 ± 12.3 y Mean disease duration: 16.8 ± 12.1 m	1 m	1. Total clinical efficacy rate 2. Neurological deficit scale 3. Barthel index 4. SF-36 score	1. *p* < 0.05 2. *p* < 0.05 3. *p* < 0.05 4. *p* < 0.05
Fan et al., 2018 (52)	Bushenyisui capsule 6#tid + CT	24 (M: 4, F: 20) Mean age: 31.63 ± 10.05 y Mean disease duration: 65.63 ± 69.85 m	26 (M: 6, F: 20) Mean age: 32.73 ± 9.84 y Mean disease duration: 43.85 ± 51.37 m	3 m	1. EDSS 2. Total clinical efficacy rate 3. Adverse events	1. *p* > 0.05 2. *p* < 0.01
Li 2017 (51)	*Tripterygium wilfordii* polyglycosides tablet 1 mg/(kg·d) bid + CT	45 (M: 15, F: 30) Mean age: 34.24 ± 5.21 y Mean disease duration: 1.24 ± 0.21 y	45 (M: 14, F: 31) Mean age: 34.21 ± 5.24 y Mean disease duration: 1.21 ± 0.25 y	3 m	1. Total clinical efficacy rate 2. Neurological Symptom Score (NSS)	1. *p* < 0.05 2. *p* < 0.05
Wu et al., 2016 (50)	Yangganyishen formula plus–minus 1 dose/d (200 mL) + CT	21 (M: 6, F: 15) Mean age: 36.8 ± 3.6 y Mean disease duration: 3 ± 1.5 y	19 (M: 7, F: 12) Mean age: 35.4 ± 4.8 y Mean disease duration: 4.3 ± 0.9 y	1 m	1. EDSS 2. Barthel index	1. *p* < 0.05 2. *p* < 0.05
Lu 2016 (49)	Self-made jieduyimian decoction 1 dose/d + CT	36 (M: 14, F: 22) Mean age: 41.2 ± 7.6 y Mean disease duration: 11.8 ± 4.6 y	36 (M: 15, F: 21) Mean age: 42.7 ± 7.2 y Mean disease duration: 12.1 ± 4.7 y	135 d	1. EDSS 2. Annual relapse frequency	1. *p* < 0.05 2. *p* < 0.01
Chen and Wang 2016 (48)	Dihuangheji 6#tid + CT	54 (M: 30, F: 24) Mean age: 66.3 ± 5.63 y Mean disease duration: N.R.	54 (M: 30, F: 22) Mean age: 68.1 ± 4.86 y Mean disease duration: N.R.	135 d	1. EDSS 2. Annual relapse frequency	1. *p* < 0.01 2. *p* < 0.05
Chen and Fan 2016 (47)	Bushenhuatan formula plus–minus 1 dose/d + CT	30 (M: 4, F: 26) Mean age: 39.2 ± 10.8 y Mean disease duration: 3.64 ± 4.17 y	30 (M: 6, F: 24) Mean age: 37.46 ± 11.09 y Mean disease duration: 4.43 ± 3.2 y	3 m	1. Multiple Sclerosis Impact Scale, MSIS - 29 2. Modified Fatigue Impact Scale, MFIS	1. *p* < 0.05 2. *p* < 0.05
Zhou and Fan 2015 (46)	Erhuang formula 1 dose/d (200 mL) + CT	43 (M: 11, F: 32) Mean age: 30.77 ± 9.82 y Mean disease duration: 2.81 ± 2.05 y	24 (M: 9, F: 15) Mean age: 36.54 ± 11.64 y Mean disease duration: 2.71 ± 1.6 y	N.R.	1. EDSS 2. Annual relapse rate 3. Annual relapse frequency	1. *p* > 0.05 2. *p* < 0.01 3. *p* < 0.01
Li et al., 2015 (45)	CHM 1 dose/d + CT	24 (N.R.) Mean age: N.R. Mean disease duration: N.R.	24 (N.R.) Mean age: N.R. Mean disease duration: N.R.	1 m	1. Total clinical efficacy rate 2. Treatment onset time 3. Average length of stay 4. Adverse events	1. *p* < 0.05 2. *p* < 0.05 3. *p* < 0.05
Zhou et al., 2013 (44)	Shuganjianpigusui formula1 dose/d (400 mL) + CT	14 (NR) Mean age: 48.86 ± 10.54y Mean disease duration: 43.36 ± 39.7 m	21 (NR) Mean age: 46 ± 10.25 y Mean disease duration: 48.38 ± 40.52 m	3 w	1. Annual relapse frequency 2. Annual relapse interval	1. *p* < 0.05 2. *p* < 0.05
Zhao 2013 (43)	Bushengusui tablet 6#tid + CT	18 (M: 9, F: 9) Mean age: 40.2 y Mean disease duration: 25 m	18 (M: 10, F: 8) Mean age: 40.5 y Mean disease duration: 22 m	3 m	1. Total clinical efficacy rate 2. EDSS 3. Average length of stay 4. Adverse events	1. *p* < 0.05 2. *p* < 0.01 3. *p* < 0.05
Wei 2012 (42)	CHM 1 dose/d + CT	25 (M: 4, F: 21) Mean age: N.R Mean disease duration: N.R	20 (M: 5, F: 15) Mean age: N.R Mean disease duration: N.R	3 m	1. Annual relapse interval	1. *p* < 0.05
Pu 2012 (41)	CHM 1 dose/d (200 mL) + CT	22 (M: 80, F: 14) Mean age: 34.5 ± 12.69 y Mean disease duration: 16.3 m	21 (M: 7, F: 14) Mean age: 38.95 ± 14.09 y Mean disease duration: 17.1 m	1 m	1. EDSS 2. Total clinical efficacy rate	1. *p* < 0.05 2. *p* < 0.05
Li and Zhao 2012 (40)	CHM decoction 1 dose/d + CT	30 (M: 12, F: 18) Mean age: 40.24 ± 2.53 y Disease duration: 3.31 ± 1.25 y	30 (M: 13, F: 17) Mean age: 42.31 ± 1.24 y Disease duration: 3.26 ± 1.08 y	3 m	1. EDSS 2. Total clinical efficacy rate	1. *p* < 0.01 2. *p* < 0.05
Yang et al., 2009 (39)	Simiaoyongan decoction and marrow storing pill plus–minus 1 dose/d	30 (M: 11, F: 19) Mean age: 35.67 ± 12.23 y Mean disease duration: N.R	15 (M: 5, F: 10) Mean age: 35.17 ± 12.64 y Mean disease duration: N.R	3 m	1. Total clinical efficacy rate	1. *p* < 0.05
Zeng et al., 2009 (38)	Buyanghuanwu decoction plus–minus 1 dose/d + CT	35 (M: 15, F: 20) Mean age: 42 y Mean disease duration: N.R	30 (M: 11, F: 19) Mean age: 40 y Mean disease duration: N.R	1 m	1. Total clinical efficacy rate 2. Adverse events	1. *p* < 0.05
Gao et al., 2008 (37)	Dihuanheji capsule 4#tid + CT	38 (M: 21, F: 17) Mean age: 37.10 ± 7.56 y Mean disease duration: N.R	40 (M: 24, F: 16) Mean age: 36.35 ± 7.67 y Mean disease duration: N.R	3 w	1. EDSS 2. Annual relapse frequency	1. *p* < 0.001 2. *p* < 0.05
Fan et al., 2007 (36)	Erhuang formula + CT	30 (M: 9, F: 21) Mean age: 38.1 ± 12.48 y Mean disease duration: N.R	35 (M: 8, F: 27) Mean age: 36.46 ± 14.13 y Mean disease duration: N.R	N.R.	1. EDSS 2. Annual relapse frequency	1. *p* < 0.05 2. *p* > 0.05
Zuo and Jia 2006 (35)	Yishengujintongluo formula 1 dose/d + CT	30 (M: 13, F: 17) Mean age: 33.4 ± 10.5 y Mean disease duration: 17.3 ± 11.8 m	30 (M: 12, F: 18) Mean age: 31.9 ± 12.3 y Mean disease duration: 16.8 ± 12.1 m	2 m	1. Total clinical efficacy rate 2. EDSS	1. *p* > 0.05 2. *p* < 0.01
Zhang and Zhang 2006 (34)	Gusuitongluo decoction 1 dose/d + CT	30 (M: 13, F: 17) Mean age: 33.41 ± 10.52 y Mean disease duration: 17.32 ± 11.82 m	30 (M: 12, F: 18) Mean age: 31.93 ± 12.31 y Mean disease duration: 16.82 ± 12.14 m	2 m	1. EDSS 2. Total clinical efficacy rate	1. *p* < 0.05 2. *p* < 0.05
Wang et al., 2006 (33)	Jiweiling decoction 1 dose/d + CT	36 (M: 20, F: 16) Mean age: 26.25 ± 6.70 y Mean disease duration: 2.58 ± 0.34 m	32 (M: 14, F: 18) Mean age: 27.65 ± 5.8 y Mean disease duration: 2.64 ± 0.41 m	2 m	1. Total clinical efficacy rate 2. Adverse events	1. *p* < 0.01
Shi and Wang 2004 (32)	Jiannaogusui decoction 1 dose/d + CT	19 (M: 7, F: 12) Mean age: 32.5 ± 11.6 y Mean disease duration: 40.5 ± 37.6 m	19 (M: 8, F: 11) Mean age: 33.1 ± 10.2 y Mean disease duration: 41.3 ± 31.53 m	3 m	1. Total clinical efficacy rate 2. EDSS 3. Annual relapse frequency 4. Adverse events	1. *p* < 0.05 2. *p* < 0.01 3. *p* < 0.01

CHM, Chinese herbal medicine; MPPT, methylprednisolone; N.R., no reported; EDSS, Expanded Disability Status Scale; CT, control therapy; d, day(s); w, week(s); m, month(s); y, year(s); tid, ter in die; bid, bis in die; ivgtt, intravenously guttae; po, per os; M, male; F, female.

Adverse effects were reported in 6 studies (32, 33, 38, 43, 45, 52), while the remaining 19 studies did not mention them. Four (32, 38, 43, 52) out of the six studies reported that no adverse effects happened in the CHM group. Wang et al. (33) reported two cases of adverse events in the CHM group and three cases in the WM group. Obesity and acne (45) were reported in the CHM group. Obesity, acne, liver dysfunction, and neuropsychiatric symptoms were reported in the WM group (43, 45). However, all studies did not mention life-threatening adverse effects.

### 3.3 Reporting quality

**Figure 2:**
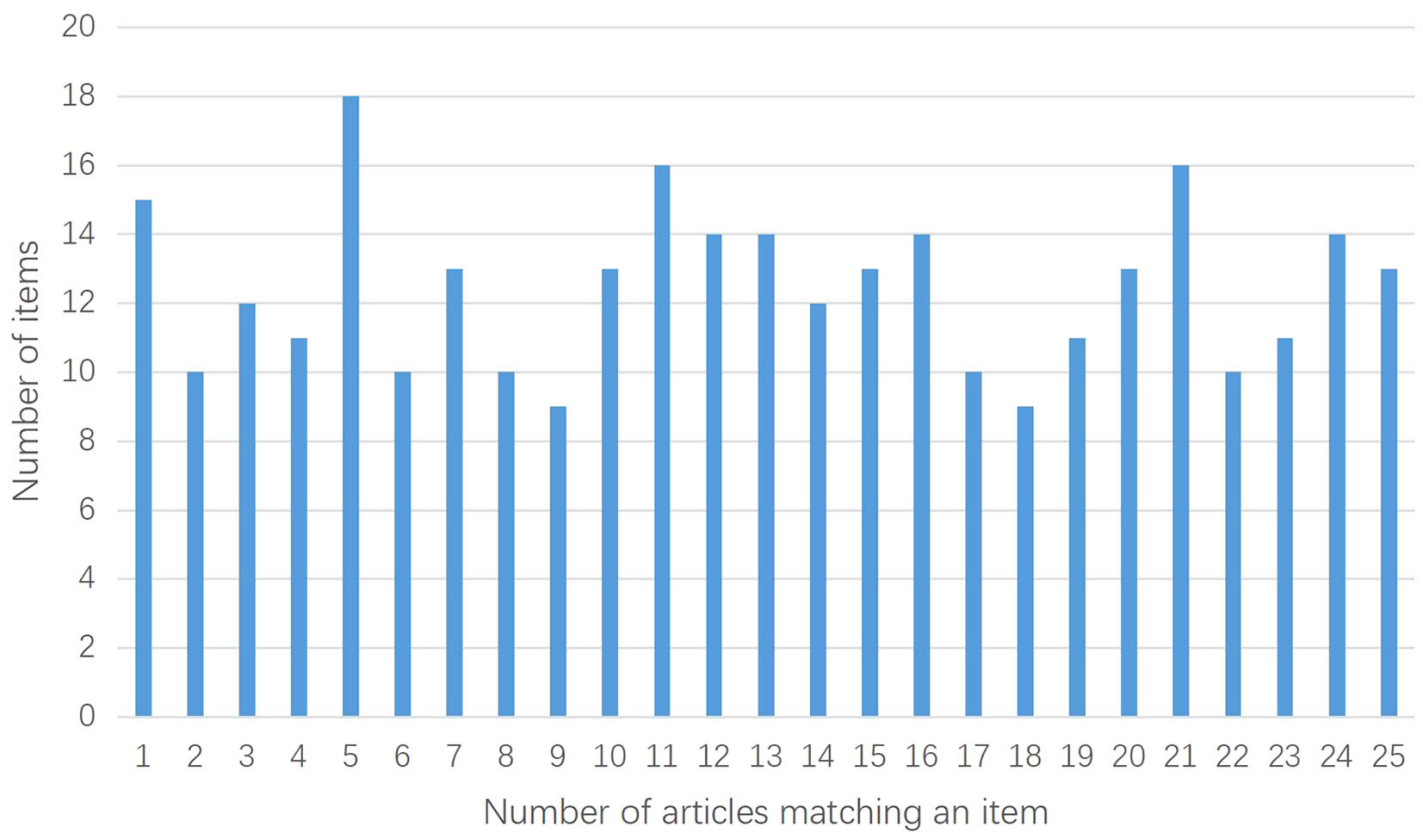
Distribution of the number of CONSORT items satisfied by the included studies.

**Figure 3:**
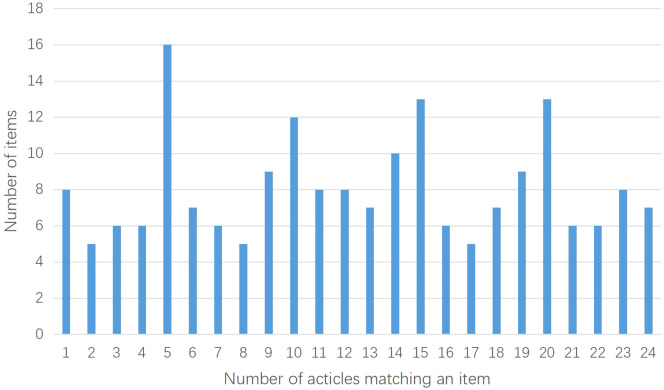
Distribution of the number of CONSORT-CHM Formulas items satisfied by the included studies.

**Figure 4:**
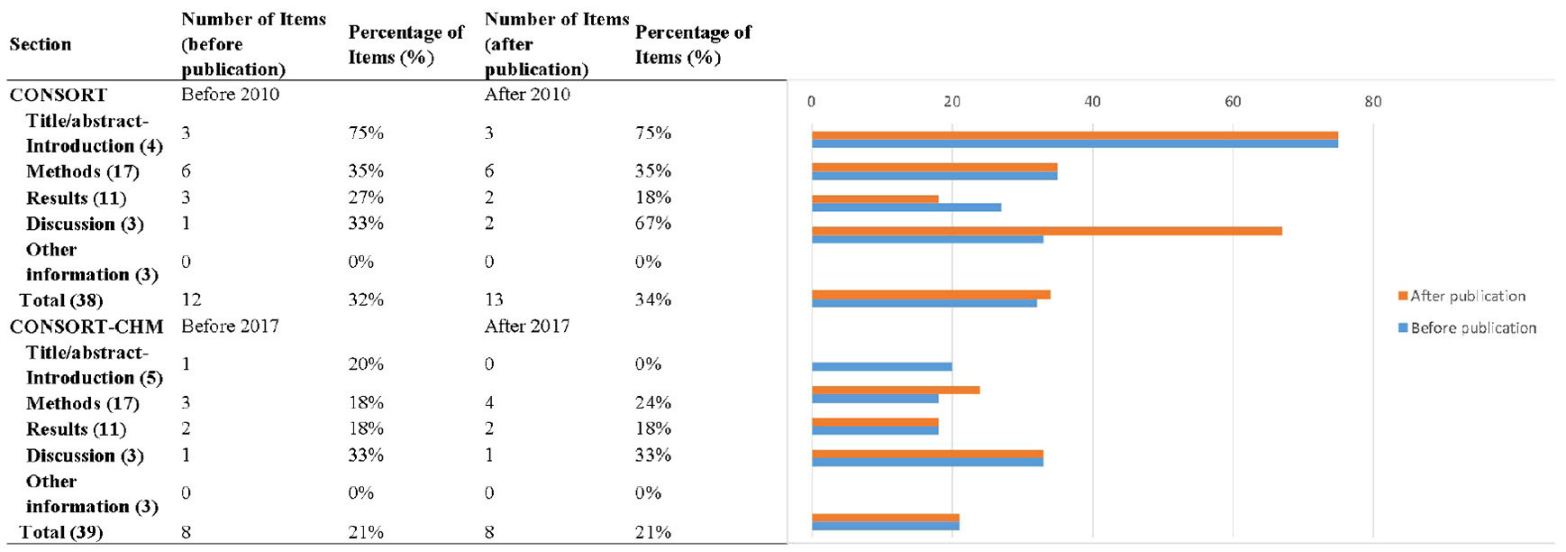
Numbers and percentages of CONSORT and CONSORT-CHM Formulas checklist sections reported by time period.

The 25 included studies consisted of 24 CHM formula studies and 1 CHM monomer study (51). The CONSORT-CHM Formulas checklist is used to evaluate the reporting quality of CHM formula RCTs. We therefore performed CONSORT scores on all included studies and CONSORT-CHM scores on 24 studies except for the monomer study. The distribution of the number of CONSORT and CONSORT-CHM Formulas items satisfied by the included studies is shown in **Figures 2**, **3**. The CONSORT and CONSORT-CHM Formulas checklist sections are summarized in **Figure 4** and individual items are described in **Table 2**.

**Table 2:** Number and percentage of CONSORT and CONSRT-CHM Formulas checklist items reported in the included studies.

Section/Topic	Item no.	Checklist item	*n*	CONSORT% (*n*/25) CONSORT% CHM (*n*/24)	95% CI
Title and abstract	1a	Identification as a randomized trial in the title	1	4%	[0 to 20]
	1a*	Statement of whether the trial targets a TCM pattern, a Western medicine-defined disease, or a Western medicine-defined disease with a specific TCM pattern	1	4%	[0 to 21]
	1b	Structured summary of trial design, methods, results, and conclusions (for specific guidance, see CONSORT for abstracts)	23	92%	[74 to 99]
	1b*	Illustration of the name and form of the formula used, and the TCM pattern applied, if applicable	2	8%	[1 to 27]
	1c*	Determination of appropriate keywords, including “Chinese herbal medicine formula” and “RCT”	0	0%	[0 to 0]
Introduction					
Background and objectives	2a	Scientific background and explanation of rationale	19	76%	[55 to 91]
	2a*	Statement with biomedical science approaches and/or TCM approaches	6	25%	[10 to 47]
	2b	Specific objectives or hypotheses	22	88%	[69 to 98]
	2b*	Statement of whether the formula targets a Western medicine-defined disease, a TCM pattern, or a Western medicine-defined disease with a specific TCM pattern	6	25%	[10 to 47]
Methods					
Trial design	3a	Description of trial design (such as parallel, factorial) including allocation ratio	24	96%	[80 to 100]
	3b	Important changes to methods after trial commencement (such as eligibility criteria), with reasons	0	0%	[0 to 0]
Participants	4a	Eligibility criteria for participants	15	60%	[39 to 79]
	4a*	Statement of whether participants with a specific TCM pattern were recruited	4	17%	[5 to 37]
	4b	Settings and locations where the data were collected	24	96%	[80 to 100]
Interventions	5	The interventions for each group with sufficient details to allow replication, including how and when they were actually administered	25	100%	[100 to 100]
	5*	Description(s) for different types of formulas should include specific contents	0	0%	[0 to 0]
Outcomes	6a	Completely defined pre-specified primary and secondary outcome measures, including how and when they were assessed	25	100%	[100 to 100]
	6a*	Illustration of outcome measures with pattern in detail	3	13%	[3 to 32]
	6b	Any changes to trial outcomes after the trial commenced, with reasons	0	0%	[0 to 0]
Sample size	7a	How sample size was determined	0	0%	[0 to 0]
	7b	When applicable, explanation of any interim analyses and stopping guidelines	0	0%	[0 to 0]
Randomization					
Sequence generation	8a	Method used to generate the random allocation sequence	8	32%	[15 to 54]
	8b	Type of randomization; details of any restriction (such as blocking and block size)	0	0%	[0 to 0]
Allocation concealment mechanism	9	Mechanism used to implement the random allocation sequence (such as sequentially numbered containers), describing any steps taken to conceal the sequence until interventions were assigned	0	0%	[0 to 0]
Implementation	10	Who generated the random allocation sequence, who enrolled participants, and who assigned participants to interventions	1	4%	[0 to 20]
Blinding	11a	If done, who was blinded after assignment to interventions (for example, participants, care providers, those assessing outcomes) and how	1	4%	[0 to 20]
	11b	If relevant, description of the similarity of interventions	0	0%	[0 to 0]
Statistical methods	12a	Statistical methods used to compare groups for primary and secondary outcomes	22	88%	[69 to 98]
	12b	Methods for additional analyses, such as subgroup analyses and adjusted analyses	0	0%	[0 to 0]
Results					
Participant flow (a diagram is strongly recommended)	13a	For each group, the numbers of participants who were randomly assigned, received intended treatment, and were analyzed for the primary outcome	0	0%	[0 to 0]
	13b	For each group, losses and exclusions after randomization, together with reasons	2	8%	[1 to 26]
Recruitment	14a	Dates defining the periods of recruitment and follow-up	23	92%	[74 to 99]
	14b	Why the trial ended or was stopped	0	0%	[0 to 0]
Baseline data	15	A table showing baseline demographic and clinical characteristics for each group	7	28%	[12 to 49]
Numbers analyzed	16	For each group, number of participants (denominator) included in each analysis and whether the analysis was by original assigned groups	21	84%	[64 to 96]
Outcomes and estimation	17a	For each primary and secondary outcome, results for each group, and the estimated effect size and its precision (such as 95% confidence interval)	0	0%	[0 to 0]
	17 b	For binary outcomes, presentation of both absolute and relative effect sizes is recommended	0	0%	[0 to 0]
Ancillary analyses	18	Results of any other analyses performed, including and analyses, distinguishing pre-specified from exploratory	0	0%	[0 to 0]
Harms	19	All important harms or unintended effects in each group (for specific guidance see CONSORT for harms)	7	28%	[12 to 49]
Discussion					
Limitations	20	Trial limitations, addressing sources of potential bias, imprecision, and, if relevant, multiplicity of analyses	6	24%	[9 to 45]
Generalizability	21	Generalizability (external validity, applicability) of the trial findings	8	32%	[15 to 54]
	21*	Discussion of how the formula works on different TCM patterns or diseases	3	13%	[3 to 32]
Interpretation	22	Interpretation consistent with results, balancing benefits and harms, and considering other relevant evidence	25	100%	[100 to 100]
	22*	Interpretation with TCM theory	24	100%	[100 to 100]
Other information					
Registration	23	Registration number and name of trial registry	0	0%	[0 to 0]
Protocol	24	Where the full trial protocol can be accessed, if available	0	0%	[0 to 0]
Funding	25	Sources of funding and other support (such as supply of drugs), role of funders	1	4%	[0 to 20]
Total mean score of CONSORT_a_			12.4 ± 2.4		
Total mean score of CONSORT-CHM_a_			8 ± 2.8		

CONSORT-CHM Formulas, CONSORT extension for Chinese herbal medicine formulas.

Items with * indicate that this item is CONSORT-CHM Formulas.

_a_Mean ± SD.

#### 3.3.1 CONSORT

Most of the items were satisfied by a few studies. Analysis of the included studies showed that, on average, 33% of the recommended items were reported. Fan et al. (52) reported the highest percentage of recommended items, at 47%, and Chen and Wang (48) and Yang et al. (39) reported the lowest percentage of recommended items, at 24%.

Only one (4%) trial could be identified as RCT after reading the title. Abstracts were structured appropriately in 92% of included studies and introductions were structured appropriately in 82%.

Seventeen items related to the methods section and were included in just 35% of included studies. Eight CONSORT items were not described in any of the articles (0%), and they were description of significant changes in the experimental method (item 3b), whether there are changes in the trial outcomes after the commencing of the experiment (item 6b), how sample size was determined (item 7a) and the explanation of any interim analysis and stopping guidelines (item 7b), the type of randomization (item 8b), the mechanism used to implement the random allocation sequence (item 9), the similarity of interventions (item 11b), and methods for additional analyses (item 12b).

The results sections included 11 items and were underreported. Overall, just 22% of included studies reported these items. None of the articles (0%) described the treatment progress with a diagram (item 13a), the reasons why the trial ended or was stopped (item 14b), the estimated effect size (item 17a), absolute or relative effect sizes (item 17b), and results of any other analyses performed (item 18).

Three items related to the discussion section, outlined study limitations, generalizability, and interpretation of results, and were reported in 44% of included studies. Information about the trial registration number, availability of the full protocol, and funding sources was reported in 0%, 0%, and 4% of included studies, respectively.

Studies published after the CONSORT checklist was revised in 2010 reported more items, although not significantly. Until 2010, 12 ± 2, or 32% of the items were reported in studies, and from 2010 onwards, 13 ± 2, or 34% were reported (*p* = 0.657, *t* = 0.449).

#### 3.3.2 CONSORT-CHM Formulas

The CONSORT-CHM Formulas checklist items were not satisfactorily reported. Of the 39 items, an average of just 21% was reported in the included studies. Fan et al. (52) reported the highest percentage of recommended items, at 41%, and Shi (55), Chen and Wang (48), and Yang et al. (39) reported the lowest percentage of recommended items, at 13%.

Abstracts need to illustrate the name and form of the formula used and the TCM pattern applied. This was reported in only two (8%) of the included studies. None of the articles determined appropriate keywords, including “Chinese herbal medicine formula” and “RCT”. Introductions satisfied the criteria in 25% of the studies.

The methods section was poorly described, with only 21% of items reported on average. Four (17%) trials stated whether participants with a specific TCM pattern were recruited. Three (13%) articles reported the outcome measures related to TCM syndrome in detail. However, none of the articles described the CHM formula in detail, especially the source and authentication method of each CHM ingredient.

Items for the results section were the same as CONSORT. Discussion sections satisfied the criteria in 46% of the studies, but only three (13%) articles provided the discussion of how the formula works on different TCM patterns on disease.

After the CONSORT-CHM Formulas was published in 2017, RCT quality did not improve significantly. Until 2017, 8 ± 2, or 21% of the items were reported in studies, and from 2017 onwards, 8 ± 4, or 21% were reported (*p* = 0.894, *t* = 0.135).

### 3.4 Risk of bias

**Figure 5:**
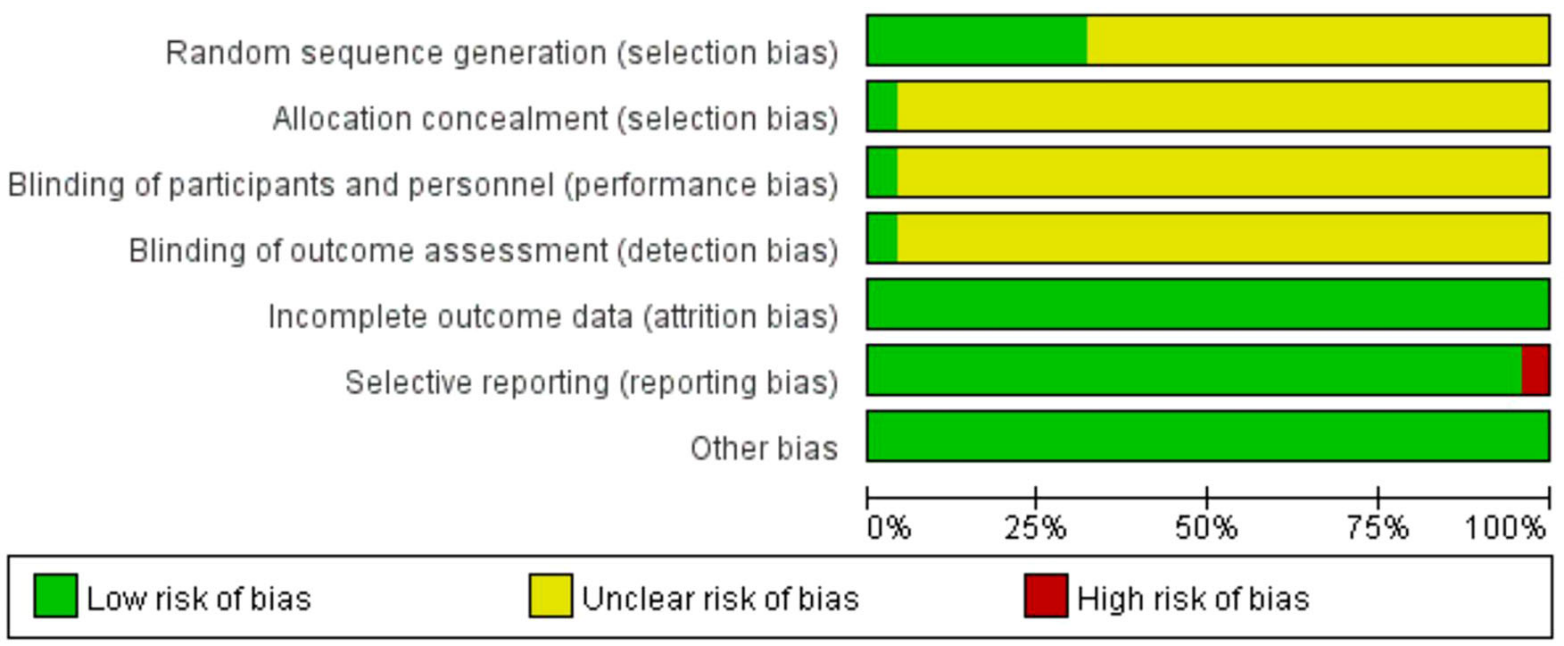
Risk of bias graph.

**Figure 6:**
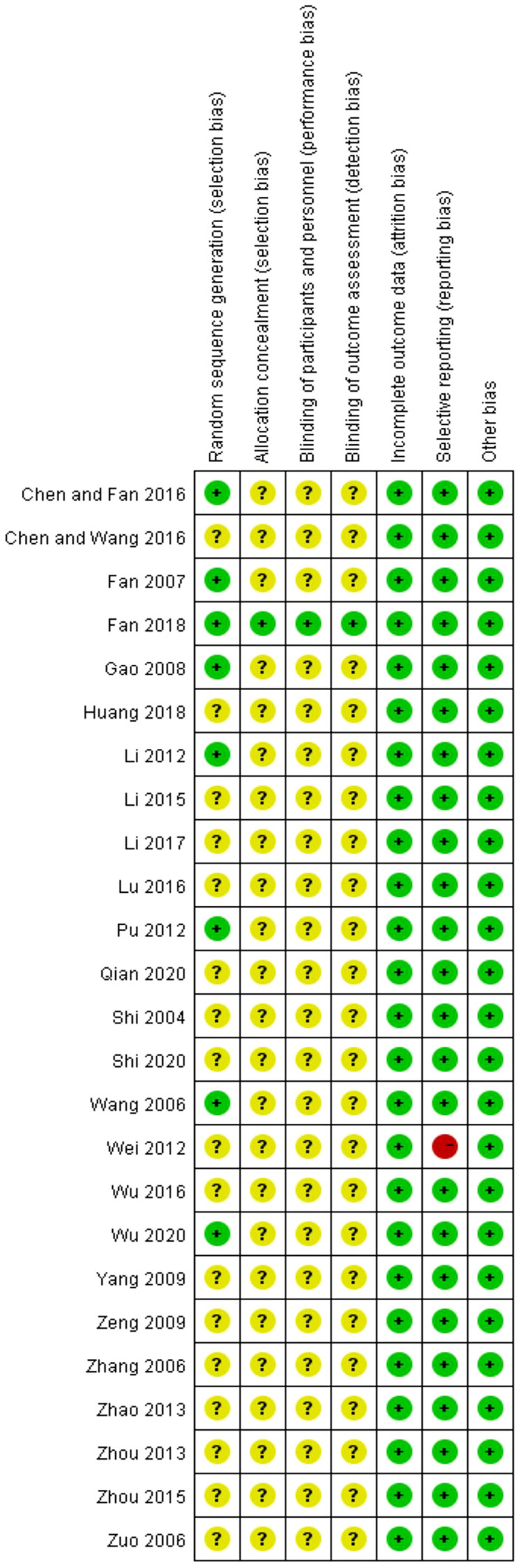
Risk of bias summary.

The summary and graph of the risk of bias are shown in **Figures 5**, **6**. Although all the included trials claimed randomization, only eight trials (33, 36, 37, 40, 41, 47, 52, 56) reported the method of random sequence generation. Only one study (52) mentioned allocation concealment, the blinding of participants, and investigator and outcome assessment. All studies reported complete outcome data. All of the studies were free of selective reporting except one study (42). All studies had low risk of other bias, which included incomparable baseline characteristics between the groups. The average item of low risk of bias for the 25 trials was 3.4, accounting for 49% of the total items.

### 3.5 Description of the CHMs

A total of 92 herbs were included in the 25 studies. The top 29 most frequently used herbs were ordinally Prepared Rehmannia Root (*Radix Rehmanniae Preparata*), Fresh Rehmannia Root (*Radix Rehmanniae Recens*), Epimrdii (*Herba Epimedii*), Scorpion (*Scorpio*), Indian Bread (*Poria*), Asiatic Cornelian Cherry Fruit (*Fructus Corni*), Stiff Silkworm (*Bombyx Batryticatus*), Root of Membranous Milkvetch (*Radix Astragali*), Root of Medicil cyathula (*Radix Cyathulae*), Root of Chinese Angelica (*Radix Angelicae Sinensis*), Root of Ural Licorice (*Radix Glycyrrhizae*), Red Peony Root (*Radix Paeoniae Rubra*), Root Pilose Asiabell (*Radix Codonopsis pilosulae*), Dodder Seed (*Semen Cuscuta*), Tuber Fleeceflower (*Polygonum Multiflorum*), Seed of Jobstears (*Semen Coicis*), White Peony Root (*Radix Paeoniae Alba*), Desertliving Cistanche (*Herba Cistanches*), Phellodendron Bark (*Cortex Phellodendri*), Earthworm (*Pheretima*), Tall Gastrodia (*Gastrodia Elata*), Common Yam Rhizome (*Rhizoma Dioscoreae*), Eucommia Bark (*Cortex Eucommiae*), Fruit of Chinese Wolfberry (*Fructus Lycii*), Dried Tangerine Peel (*Pericarpium Citri Reticulatae*), Bulb of Fritillary (*Bulbus Fritillariae*), Leech (*Hirudo*), Szechuan Lovage Root (*Rhizoma Chuanxiong*), and Tuber of Pinellia (*Rhizoma Pinelliae*), which were used more than four times (**Table 3**). The cumulative percentage of these herbs was 62%.

**Table 3:** Analysis of high-frequency herbs in the treatment of MS.

Chinese name	English name	Latin name	Frequency	The total frequency%	Cumulative percentiles%
Shudihuang	Prepared Rehmannia root	*Radix Rehmanniae Preparata*	11	4%	4%
Shengdihuang	Fresh Rehmannia root	*Radix Rehmanniae Recens*	9	4%	4%
Yinyanghuo	Epimrdii	*Herba Epimedii*	8	3%	3%
Quanxie	Scorpion	*Scorpio*	8	7%	7%
Fuling	Indian bread	*Poria*	8	3%	3%
Shanzhuyu	Asiatic cornelian cherry fruit	*Fructus Corni*	8	10%	10%
Jiangcan	Stiff silkworm	*Bombyx Batryticatus*	7	3%	3%
Huangqi	Root of membranous milkvetch	*Radix Astragali*	7	13%	13%
Chuanniuxi	Root of Medicil cyathula	*Radix Cyathulae*	7	3%	3%
Danggui	Root of Chinese Angelica	*Radix Angelicae Sinensis*	7	16%	16%
Gancao	Root of ural licorice	*Radix Glycyrrhizae*	6	3%	3%
Chishao	Red peony root	*Radix Paeoniae Rubra*	6	29%	29%
Dangshen	Root of pilose asiabell	*Radix Codonopsis pilosulae*	6	3%	3%
Tusizi	Dodder seed	*Semen Cuscuta*	6	18%	18%
Heshouwu	Tuber fleeceflower	*Polygonum Multiflorum*	6	3%	3%
Yiyiren	Seed of jobstears	*Semen Coicis*	6	21%	21%
Baishao	White peony root	*Radix Paeoniae Alba*	5	2%	44%
Roucongrong	Desertliving cistanche	*Herba Cistanches*	5	2%	46%
Huangbo	Phellodendron bark	*Cortex Phellodendri*	5	2%	47%
Dilong	Earthworm	*Pheretima*	4	1%	49%
Tianma	Tall gastrodia	*Gastrodia elata*	4	1%	50%
Shanyao	Common yam rhizome	*Rhizoma Dioscoreae*	4	1%	52%
Duzhong	Eucommia bark	*Cortex Eucommiae*	4	1%	53%
Gouqizi	Fruit of Chinese wolfberry	*Fructus Lycii*	4	1%	55%
Chenpi	Dried tangerine peel	*Pericarpium Citri Reticulatae*	4	1%	56%
Beimu	Bulb of fritillary	*Bulbus Fritillariae*	4	1%	58%
Shuizhi	Leech	*Hirudo*	4	1%	59%
Chuanxiong	Szechuan lovage root	*Rhizoma Chuanxiong*	4	1%	61%
Banxia	Tuber of pinellia	*Rhizoma Pinelliae*	4	1%	62%

The summary of the mechanisms of Chinese medicine monomers in the treatment of MS is shown in **Table 4**. Most Chinese medicine monomers can relieve inflammatory injury by inhibiting PI3K/Akt, NF-κB, and TLR4 signaling pathways and reducing the release of inflammatory factors TNF-α, IL-17A, IL-6, IL-23, IL-1β, and IFN-γ (58, 65, 68). Ginsenoside-Rg3 can regulate NOX2/4 oxidative stress-related pathways, reduce the expression of NOX, exert the effect of anti-oxidative stress, and improve the blood–brain barrier integrity (64). Ginsenoside Rd, Tanshinon IIA, Cordyceps sinensis extract, Rhodiola rosea, Baicalein, and Periplocoside A regulate the imbalance of Th17/Th1/Treg subsets and modulate immunity (63, 67, 70, 71, 73). Emodin and Matrine can upregulate the expression of the neurotransmitter-nutrient factor BDNF, improve the microenvironment of nerve survival, promote myelin repair and regeneration, and exert neuroprotective effects (65, 69).

**Table 4:** The mechanisms of Chinese medicine monomers in the treatment of MS.

English name of Chinese medicine	Latin name	Monomer	Dosage	Treatment duration	Mechanism of action
Rehmannia glutinosa Libosch	*Radix Rehmanniae Preparata*	Catalpol	10 mg/kg	Injected intraperitoneally 40 d	Increase tyrosine hydroxylase expression and noradrenaline levels, neuroprotective effect (57)
Epimrdii	*Herba Epimedii*	Icariin	25 mg/kg	Administered intragastrically 42 d	Reduce AKT, iNOS, TNF-α, TGF-β1, and NF-κB, inhibition of oxidative stress and inflammatory response (58)
Asiatic cornelian cherry fruit	*Fructus Corni*	Cornuside	150 mg/kg/d	Administered intragastrically 28 d	Reduce IL-17A, IL-6, and IL-23, anti-inflammatory and immunosuppressive effects by inhibiting Th17 cells (59)
Root of membranous milkvetch	*Radix Astragali*	Total flavonoids of Astragalus	25 and 50 mg/kg/d	Administered intragastrically 21 d	Reduce NO, TNF-α, IL-6, and IL-1β, inhibition of microglia-mediated inflammation (60)
		Astragaloside IV	20 mg/kg	Injected intraperitoneally 10 d	Suppress the maturation and function of dendritic cells (13)
Root of ural licorice	*Radix Glycyrrhizae*	Glycyrrhizin	10, 25, and 50 mg/kg/d	Injected intraperitoneally 23 d	Reduce TNF-α, IFN-γ, IL-17A, and IL-6, and increase IL-4, inhibition of inflammatory response (61)
White peony root	*Radix Paeoniae Alba*	Total glucosides of paeony	0.2 g/kg, 0.4 g/kg	Administered intragastrically 14 d	Reduce mTOR and HIF-1α, anti-inflammatory and immune regulation (62)
Ginseng	*Radix Ginseng*	Ginsenoside Rd	20, 40, and 80 mg/kg	Administered intragastrically 21 d	Reduce IFN-γ, IL-6, and IL-10, and regulate the imbalance of Th17/Th1/Treg, anti-inflammatory, and immune regulation (63)
Red ginseng	*Radix Ginseng Rubra*	Ginsenoside-Rg3	500 mg/kg	Administered intragastrically 33 d	Reduce NOX2 and NOX4 expression to improve the blood–brain barrier integrity, neuroprotective, anti-inflammatory, and anti-oxidative effects (64)
Root and rhizome of sorrel rhubarb	*Radix et Rhizoma Rhei*	Emodin	30 and 60 mg/kg/d	Administered intragastrically 21 d	Increase MBP and BDNF. Reduce p-Akt, p-PI3K, and NF-κB, inhibition of microglial activation, anti-inflammatory, and neuroprotective effects (65)
Tarragon	*Artemisia dracunculus*	Artemisia dracunculus extracts	500 mg/kg/d	Administered intragastrically 33 d	Reduce IL-17 and IL-23, inhibition of inflammatory effect (66)
Root of ligulilobe sage	*Radix Salviae Liguliobae*	Tanshinon IIA	25 μmol/L	Injected intraperitoneally 11d	Reduce the demyelination and the number of inflammatory cells, and increase regulatory T cells (67)
Common threewingnut root	*Radix Tripterygii Wilfordii*	Tripterygium glycosides	9 mg/kg	Administered intragastrically 5 d	Reduce TLR4, TLR9, TNF-α, IL-1β, and IL-6, anti-inflammatory and immune regulation (68)
Root of lightyellow sophora	*Radix Sophorae Flavescentis*	Matrine	200 mg/kg/d	Injected intraperitoneally 23 d	Increase MBP and PLP, reduce p-PI3K, p-Akt, p-mTOR, and promote oligodendrocyte differentiation and myelination (69)
Chinese caterpillar fungus	*Cordyceps*	Cordyceps sinensis extract	1 g/kg, 5 g/kg	Administered intragastrically 30 d	Reduce the number of Th1 cells, inflammatory infiltration, and demyelination (70)
		Cordycepin (3’-deoxyadenosine)	50 mg/kg	Injected intraperitoneally 21 d	Reduce IFN-γ, IL-6, TNF-α, and IL-17, inhibit leukocyte infiltration and dendritic cell activation and migration, and reduce neuroinflammation
Grass of rhodiola	*Radix et Rhizoma Rhodiolae*	Rhodiola rosea	50, 100, and 200 mg/kg	Administered intragastrically 28 d	Regulate Th17/Th1 and Th17/Treg ratios, immune regulation (71)
Scutellarin baicalensis	*Radix Scutellariae*	Baicalein	25 mg/kg	Administered intragastrically 21 d	Suppress pathogenetic CXCR6 CD4 cells ^+^, reduce IL-17A production, and inhibit Th17 differentiation (72)
Periploca sepium Bge	*Periploca nigrescens*	Periplocoside A	25 and 50 mg/kg	Administered intragastrically 25 d	Suppress IL-17 production and the differentiation of Th17 cells, immunosuppressive and anti-inflammatory effects (73)

## 4 Discussion

### 4.1 Principal findings

This article revealed that less than 50% of CONSORT and CONSORT-CHM Formulas items were reported in RCTs of CHM for MS. Moreover, revision of the CONSORT checklists and publication of CONSORT-CHM Formulas checklists had not significantly improved the quality of reporting in these studies. Consistently, the risk of bias in most RCTs was classified as “unclear.” The low proportion of CONSORT items reported and the unclear risk of bias indicated that the quality of RCTs of CHM for MS was inadequate in terms of completeness of reporting and validity of results.

#### 4.1.1 Reporting quality

RCTs of CHM in the treatment of MS had poor compliance with CONSORT and CONSORT-CHM Formulas statements. The average CONSORT and CONSORT-CHM Formulas proportions of all included studies were only 33% and 21%, respectively. To sum up, these studies had the following shortcomings:

From the perspective of CONSORT (1): The title did not indicate that the corresponding article is an RCT. Only one article in this study could be seen as an RCT based on the title (2). No article explained how to calculate the sample size. Relevant studies have found that if the pre-test sample size is not estimated, there is a lack of statistical ability to ensure the proper estimation of the treatment effect (74) (3). Most articles lacked the description of the randomization process, allocation concealment, and the blind method. However, randomization is necessary to avoid selection bias and the blinding procedure is an essential method for preventing research outcomes from being influenced by either the placebo effect or observer bias (4). No article showed a diagram of participant flow. Dropouts were only reported in one trial. These might reduce the credibility of the results (5). The estimated effect size or absolute and relative effect size can help readers better understand the benefits of drugs, but no article in this study provided the corresponding content (6). In the discussion, only a few articles explained the limitations and generalizability (7). Among other things, none of the trials in this study were registered and provided trial protocol. It was not conducive to improve transparency and accountability (75). Only one article explained the source of funds and conflict of interest.

From the perspective of CONSORT-CHM Formulas (1): No article added “Chinese herbal medicine formula” and “RCT” to the keywords (2). None of the articles reported in detail the source and authentication method of each CHM ingredient of CHM formula (3). All articles described the outcome indicators in detail, but only 13% of them reported the outcome indicators related to traditional Chinese medicine (TCM) syndromes.

The poor reporting quality of RCTs related to CHMs may not only affect the judgment of commentators and readers on their efficacy and safety (76) and reduce the value of CHMs, but also finally hinder the application and development of CHMs in the treatment of MS. Using the CONSORT guideline to ensure complete reporting is not difficult, and even brief additions during manuscript writing can resolve important omissions. For example, merely adding the words “random” or “randomized” to the title increases the likelihood that readers can readily identify RCTs (77). Although blinding itself may not always be feasible, reporting the extent to which blinding was addressed is crucial to enable readers to judge whether the presence or absence of blinding may have influenced results. Registration of study protocols allows readers to contrast planned and reported study methods, assists researchers in identifying ongoing work, and reduces unnecessary duplication (78). Furthermore, descriptions of detailed information about the herb would help readers to generalize and replicate the study (79). If reporting guidelines such as CONSORT-CHM Formulas are used at the outset of study design, the impact on reporting quality may be the greatest.

#### 4.1.2 Association and comparison between the risk of bias and reporting quality

In this article, we found that the study with the highest percentage of CONSORT and CONSORT-CHM Formulas items had the lowest risk of bias. Studies in other medical fields showed similar findings to our study, in that better reporting quality was associated with lower risk of bias (80–82). However, the study with the lowest percentage of CONSORT and CONSORT-CHM Formulas items did not have the worst performance in the risk of bias. This indicated that although studies with poor reporting quality were incomplete and had low repeatability, the risk of bias might not be high and the results were valid to some extent. In this article, the study with the highest risk of bias did not have the lowest percentage of CONSORT and CONSORT-CHM Formulas items. It showed that the study with a high risk of bias lacked description of random sequence generation, allocation concealment, and outcome assessment blinding, but this did not mean the authors of the study used inappropriate research methods. Therefore, reporting quality and risk of bias are completely different concepts and still appear to create confusion in how they are being applied in the biomedical literature (26).

The risk of bias assesses the internal validity of RCTs, in other words, an evaluation of the true effect estimate. The reporting quality assesses the completeness of reporting. The risk of bias tool is most useful for assessing the authenticity of the result and bias retrospectively, but the CONSORT checklists are more appropriately applied in the guidance of prospective clinical studies (79). If researchers expect to obtain more complete and transparent RCTs in retrospective studies, the CONSORT checklists can also be a good choice.

It is worth noting that the herbal compounds are often in their natural form and quality control including authentication of constituents can be variable (83). Furthermore, effective application of traditional medicine theory to ensure data are valid and can be properly interpreted is difficult (84). The CONSORT-CHM Formulas adequately take into account the unique characteristics of TCM—theory, principles, formulas, and Chinese medicinal substances (24), which offer tailored guidance for assessing the methodological rigor and transparency of RCTs in CHM formulas. However, despite the availability of the CONSORT-CHM Formulas, introduced in 2017, its utilization appears to be less widespread compared to other CONSORT extensions (85). CONSORT extensions for nonpharmacological treatments have proven valuable in evaluating the reporting quality of relevant RCTs in corresponding domains (86). In this article, we recognized the potential of CONSORT-CHM Formulas in improving the reporting quality of RCTs in CHM formulas. Therefore, to develop better-quality RCTs of CHM for MS, a rigorous design that integrates safety, efficacy, and patient-centered endpoints in accordance with the CONSORT-CHM Formulas guidelines is needed.

### 4.2 CHM in the treatment of MS

In the included studies, there were seven (33, 36, 37, 41, 47, 52, 55) studies with a low bias score of 4 and above, and CONSORT and CONSORT-CHM scores of 8 and above, which were of higher quality in terms of result validity and reporting completeness. A total of 33 herbs were used in these seven studies. Prepared Rehmannia Root (*Radix Rehmanniae Preparata*) and Fresh Rehmannia Root (*Radix Rehmanniae Recens*) were used five and four times, respectively, and were also the top two herbs in our count of high-frequency herbs. The remaining seven herbs that were used three times were as follows: Scorpion (*Scorpio*), Tuber Fleeceflower (*Polygonum Multiflorum*), Bulb of Fritillary (*Bulbus Fritillariae*), Leech (*Hirudo*), Motherwort (*Herba Leonuri*), Tall Gastrodia (*Gastrodia Elata*), and Weeping Forsythia Capsule (*Fructus Forsythiae*), all of which were also high-frequency herbs, except for Motherwort and Weeping Forsythia Capsule. The seven studies reported four outcome measures. Four (36, 37, 41, 52) studies used EDSS, and three (36, 37, 41) studies showed a significant effect of CHM in reducing EDSS compared to the WM group. Four (33, 41, 52, 55) studies reported that CHM significantly improved the total clinical efficacy rate. One (33) out of two (33, 36) studies reported that CHM significantly reduced the annual relapse frequency. One (55) study reported that CHM significantly reduced the annual relapse rate. The selected high-frequency herbs from the present study mostly tonified the liver and kidney. In TCM, it is widely believed that the pathogenesis of MS is related to deficiencies in the kidney, liver, and spleen. These high-frequency herbs are promising candidates for future clinical applications and MS trials.

The major challenge in the treatment of MS remains understanding and targeting the continuous neurodegeneration in people with MS at present (87). The mechanisms that lead to neurodegeneration in MS involve a complex interplay between neuroinflammation, oxidative stress and mitochondrial dysfunction, and iron toxicity (88). Chinese medicine monomers have significant biological activity proved by many experiments. They can act on multiple aspects of the pathogenesis of MS, such as inflammatory response, immunity, apoptosis, and nerve injury (89). Our summary of previous research mechanisms reveal the following: The Chinese medicine monomers can reduce the release of inflammatory factors and relieve inflammatory injury by inhibiting PI3K/Akt, NF-κB, and TLR4 signaling pathways (58, 65, 68). They can also reduce NOX2 and NOX4 expression to exert the effect of anti-oxidative stress (64). The Chinese medicine monomers regulate the imbalance of Th17/Th1/Treg subsets and modulate immunity (63, 67, 70, 71, 73). The monomers can upregulate the expression of the neurotransmitter-nutrient factor BDNF and promote myelin repair and regeneration (65, 69). However, the current research mechanisms of Chinese medicine monomers are mostly limited to the study of inflammatory factors, chemokines, and common inflammatory metabolic pathways. There are few studies that discuss the potential molecular mechanisms of promoting myelin regeneration. The research on the internal relationship between various factors and the crosstalk mechanism between multiple signaling pathways is relatively scarce. Furthermore, the latest progress of MS reported in *The Lancet Neurology* journal states that “oxidative stress and mitochondrial dysfunction contributing to glial and neuronal injury, axonal energy failure, and loss of neuronal network function may be key molecular mechanisms driving disease progression. Excessive iron deposition in CNS parenchyma has been hypothesized to be a source of oxidative stress in MS (90).” Chinese medicine monomers are less studied in this area and need to be further explored.

The safety of CHM therapy for MS remains inconclusive. In this study, 24% (6/25) of RCTs mentioned the safety of interventions or investigated adverse effects. In four out of the six studies, it was reported that no adverse effects occurred in the CHM group. In the remaining studies, the adverse effects observed in the CHM group were fewer compared to the WM group. Studies have reported that herbal medicines are generally well tolerated, with adverse effects being limited to mild to moderate (91). Some herbal supplementations did not report risks of drug interactions with conventional MS drugs (4). There is still a need for powerful real-world evidence regarding the safety of CHM and more attention should be given to both recording and reporting the adverse effects of CHM therapy.

### 4.3 Strengths and limitations

To our knowledge, this is the first time the CONSORT, CONSORT-CHM Formulas checklists, and risk of bias have been simultaneously used to assess the quality of RCTs of CHM for MS.

MS remains one of the most common causes of neurological disability in the young adult population (87). A definitive curative treatment of MS is currently unavailable. The prolonged therapy of drugs constantly results in resistance and side effects. CHM formulas may provide a completely new area for the management of MS. Evaluating the quality of RCTs in CHM formulas helps bridge the gap between MS treatments using CHM formulas and modern WM. Our research may help provide a more rigorous understanding of CHM’s efficacy and safety by evaluating the quality of RCTs in CHM formulas for MS. Furthermore, in the modern era of evidence-based practice, our findings will help improve the quality of RCT in CHM formulas for MS, aiding clinicians in making informed decisions of CHM formulas based on the best available evidence. Finally, we screened high-frequency herbs as promising candidates for future clinical applications and MS trials. Mechanistic studies of Chinese medicine monomers in the field of oxidative stress, mitochondrial dysfunction, iron toxicity, and remyelination are rare and worthy of further research.

This study had some limitations. First, the number of RCTs included in this study was not enough, and there might be a risk of bias. Second, for many elements of reporting checklists and risk for bias, inconsistent judgment may have arisen from raters’ different understanding or difficulties in discerning information from the published reports. Third, the 25 included studies reported different CHMs with variation in terms of composition, dosage, and duration of interventions. Different concentration ratios of the components of CHMs produced different effects, which could influence the bias of the research. This makes it difficult to recommend specific CHMs for clinics. Fourth, exact diagnostic criteria for patients with MS were not considered, as multiple diagnostic criteria were reported in the included RCTs. Different inclusion criteria of the participants may contribute to the heterogeneity of the included studies.

## 5 Conclusions

The low reporting of CONSORT items and unclear risk of bias indicate inadequate quality of RCTs in terms of reporting completeness and result validity. The CONSORT-CHM Formulas appropriately consider the unique characteristics of CHM, including principles, formulas, and Chinese medicinal substances. To improve the quality of RCTs on CHM for MS, researchers should adhere more closely to CONSORT-CHM Formulas guidelines and ensure comprehensive disclosure of all study design elements. High-frequency herbs are promising candidates for future clinical applications and MS trials.

## Data availability statement

The original contributions presented in the study are included in the article/**Supplementary Material**. Further inquiries can be directed to the corresponding author.

## Ethics statement

This article is a literature study of previously published data and therefore does not require ethical approval or consent procedures. The manuscript does not contain any personal data in any form (including personal details, pictures or videos).

## Author contributions

J-YW: Data curation, Formal analysis, Writing – original draft, Writing – review & editing. J-LY: Data curation, Writing – original draft. J-LH: Data curation, Writing – original draft. SX: Formal analysis, Writing – review & editing. X-JZ: Formal analysis, Writing – review & editing. S-YQ: Formal analysis, Writing – review & editing. M-LC: Formal analysis, Writing – review & editing. MA: Formal analysis, Writing – review & editing. JZ: Formal analysis, Writing – review & editing. ZZ: Formal analysis, Writing – review & editing. G-QZ: Formal analysis, Funding acquisition, Writing – review & editing.
