# Supplementary material for: Reporting quality and risk of bias of randomized controlled trials of Chinese herbal medicine for multiple sclerosis
Source: Front Immunol. 2024 Aug 19;15:1429895. doi: 10.3389/fimmu.2024.1429895 (PMC11369894; doi:10.3389/fimmu.2024.1429895)
Supplement: Supplementary file 1 [file Table1.docx]

**Additional file 1.** The detailed characteristics of the included trials

| Study | Diagnosis criteria | Trial | Control | Trial  (male/female; age;Duration) | Control  (male/female; age;Duration) | Treatment during | Follow-up peried | | Outcome measure | Intergroup Differences |
| --- | --- | --- | --- | --- | --- | --- | --- | --- | --- | --- |
| Wu et al. 2020(1) | McDonald | Yangganyishen formula plus-minus 1 does/d(200 ml) + CT | Prednisone 30 mg/d po | 22(M:9,F:13)  Mean age:  36.80 y  Mean disease  duration:  3.00 ± 1.50 y | 23(M:10,F:13)  Mean age:  35.40 y  Mean disease  duration:  4.30 ± 0.90 y | 1m | | N.R. | 1. EDSS  2. Total clinical efficacy rate  3. SF-36 score | 1. *P* < 0.05  2. *P* < 0.05  3. *P* < 0.05 |
| Shi 2020(2) | Poser | Improved pingfu decoction 2-3 does/w(150-200ml) + CT | Dexamethasone + 10%Glu 500ml ivgtt for 7d + Prednisone 40 mg/d po reduced weekly 5 mg/d | 35(M:18,F:17)  Mean age:  31.49 ± 2.06 y  Mean disease  duration:  4.01 ± 1.20 y | 35(M:15,F:20)  Mean age:  32.22 ± 2.15y  Mean disease  duration:  3.58 ± 1.21 y | 1m | | N.R. | 1. Total clinical efficacy rate  2. Annual relapse rate | 1. *P* < 0.01  2. *P* < 0.05 |
| Qian and Wang 2020(3) | McDonald | Ziyinguben granule 6g/tid + CT | MPPT 1000 mg/d + 50 g/L Glu 250ml ivgtt for 3d gradully halved to 60 mg/d + Prednisone po + Danhong 30 ml/d ivgtt + citicoline injection 0.5 g/d ivgtt + calcium carbonate D3 tablet 600 mg/d po + potassium chloride sustained-release tablet 2 g/d po | 30(M:12,F:18)  Mean age:  33.47 ± 11.15 y  Mean disease  duration:  19.38 ± 7.12 m | 30(M:10,F:20)  Mean age:  34.07 ± 10.92 y  Mean disease  duration:  19.41 ± 6.11 m | 27d | | N.R. | 1. EDSS  2. Total clinical efficacy rate  3. Neurological deficit scale | 1. *P* < 0.05  2. *P* < 0.05  3. *P* < 0.05 |
| Huang et al.2018(4) | Poser | Bushentianjing formula 1 does/d(400ml) + CT | MPPT 0.5-1 g/d ivgtt for 3-5d gradully reduced to 120 mg/d + Prednisone 60 mg/d reduced 10 mg/d every 5d po | 21(M:9,F:12)  Mean age:  43.4 ± 10.5 y  Mean disease  duration:  17.3 ± 11.8 m | 21(M:10,F:11)  Mean age:  41.9 ± 12.3 y  Mean disease  duration:  16.8 ± 12.1 m | 1m | | N.R. | 1. Total clinical efficacy rate  2. Neurological deficit scale  3. Barthel index  4. SF-36 score | 1. *P* < 0.05  2. *P* < 0.05  3. *P* < 0.05  4. *P* < 0.05 |

**Table 1** (Continued)

| Study | Diagnosis criteria | Trial | Control | Trial  (male/female; age;Duration) | Control  (male/female; age;Duration) | Treatment during | Follow-up peried | Outcome measure | Intergroup Differences |
| --- | --- | --- | --- | --- | --- | --- | --- | --- | --- |
| Fan et al. 2018(5) | McDonald | Bushenyisui capslue 6#tid + CT | N.S. | 24(M:4,F:20)  Mean age:  31.63 ± 10.05 y  Mean disease  duration:  65.63 ± 69.85 m | 26(M:6,F:20)  Mean age:  32.73 ± 9.84 y  Mean disease  duration:  43.85 ± 51.37 m | 3m | N.R. | 1. EDSS  2. Total clinical efficacy rate  3. Adverse events | 1. *P*＞ 0.05  2. *P* < 0.01 |
| Li 2017(6) | McDonald | Tripterygium wilfordii polyglycosides Tablet 1mg/(kg·d) bid + CT | MPPT 500-1000 mg/d for 5d ivgtt + Prednisone 60-80 mg/d for 7d reduced 10 mg/d every 5d po | 45(M:15,F:30)  Mean age:  34.24 ± 5.21 y  Mean disease  duration:  1.24 ± 0.21 y | 45(M:14,F:31)  Mean age:  34.21 ± 5.24 y  Mean disease  duration:  1.21 ± 0.25 y | 3m | N.R. | 1. Total clinical efficacy rate  2. Neurological Symptom Score ( NSS ) | 1. *P* < 0.05  2. *P* < 0.05 |
| Wu et al.2016(7) | McDonald | Yangganyishen formula plus-minus 1 does/d(200 ml) + CT | Prednisone 30 mg/d po | 21(M:6,F:15)  Mean age:  36.8 ± 3.6 y  Mean disease  duration:  3 ± 1.5 y | 19(M:7,F:12)  Mean age:  35.4 ± 4.8 y  Mean disease  duration:  4.3 ± 0.9 y | 1m | 3m | 1. EDSS  2. Barthel index | 1. *P* < 0.05  2. *P* < 0.05 |
| Lu 2016(8) | McDonald | Self-made jieduyimian decoction 1 does/d + CT | MPPT 1000 mg/d + 5%Glu 500 ml ivgtt for 5d + Prednisone 80 mg/d for 7d reduced 10 mg/d every 5d po + MTX 7.5 mg/w po | 36(M:14,F:22)  Mean age:  41.2 ± 7.6 y  Mean disease  duration:  11.8 ± 4.6 y | 36(M:15,F:21)  Mean age:  42.7 ± 7.2 y  Mean disease  duration:  12.1 ± 4.7 y | 135d | N.R. | 1. EDSS  2. Annual relapse frequency | 1. *P* < 0.05  2. *P* < 0.01 |

**Table 1** (Continued)

| Study | Diagnosis criteria | Trial | Control | Trial  (male/female; age;Duration) | Control  (male/female; age;Duration) | Treatment during | Follow-up peried | Outcome measure | Intergroup Differences |
| --- | --- | --- | --- | --- | --- | --- | --- | --- | --- |
| Chen and Fan 2016(9) | McDonald | Bushenhuatan formula plus-minus 1 does/d + CT | Prednisone 60 mg/d reduced weekly 5 mg/d po | 30(M:4,F:26)  Mean age:  39.2 ± 10.8 y  Mean disease  duration:  3.64 ± 4.17 y | 30(M:6,F:24)  Mean age:  37.46 ± 11.09 y  Mean disease  duration:  4.43 ± 3.2 y | 3m | N.R. | 1. Multiple Sclerosis Impact Scale, MSIS － 29  2. Modified Fatigue Impact Scale, MFIS | 1. *P* < 0.05  2. *P* < 0.05 |
| Chen and Wang  2016(10) | McDonald | Dihuangheji 6#tid + CT | MPPT 500-1000 mg/d + Glu 500 ml ivgtt for 5d + Prednisone 20-50 mg/d po | 54(M:30,F:24)  Mean age:  66.3 ± 5.63 y  Mean disease  duration:N.R. | 54(M:30,F:22)  Mean age:  68.1 ± 4.86 y  Mean disease  duration:N.R. | 135d | 1y | 1. EDSS  2. Annual relapse frequency | 1. *P* < 0.01  2. *P* < 0.05 |
| Li et al.2015(11) | McDonald | CHM 1 dose/d + CT | MPPT 500-1000 mg/d + 5%Glu 500 ml ivgtt for 5d + Prednisone 60 mg/d reduced weekly 10-20 mg/d po + Vitamin B1 + Vitamin B12 po | 24(N.R.)  Mean age:N.R.  Mean disease  duration:N.R. | 24(N.R.)  Mean age:N.R.  Mean disease  duration:N.R. | 1m | N.R. | 1. Total clinical efficacy rate  2. Treatment onset time  3. Average  Length of Stay  4. Adverse events | 1. *P* < 0.05  2. *P* < 0.05  3. *P* < 0.05 |
| Zhou and Fan 2015(12) | McDonald | Erhuang formula 1 does/d(200 ml) + CT | MPPT 1000 mg/d gradually halved every 3d to 120 mg/d + Prednisone  60 mg/d | 43(M:11,F:32)  Mean age:  30.77±9.82 y  Mean disease  duration:  2.81±2.05 y | 24(M:9,F:15)  Mean age:  36.54±11.64 y  Mean disease  duration:  2.71±1.6 y | N.R. | 2y | 1. EDSS  2. Annual relapse rate  3. Annual relapse frequency | 1. *P* > 0.05  2. *P* < 0.01  3. *P* < 0.01 |

**Table 1** (Continued)

| Study | Diagnosis criteria | Trial | Control | Trial  (male/female; age;Duration) | Control  (male/female; age;Duration) | Treatment during | Follow-up peried | Outcome measure | Intergroup Differences |
| --- | --- | --- | --- | --- | --- | --- | --- | --- | --- |
| Zhao  2013(13) | McDonald | Bushengusui Tablet  6#tid + CT | MPPT 1000 mg/d for  3–5d +Prednisone  60–580 mg/d | 18(M:9,F:9)  Mean age: 40.2 y  Mean disease  duration:  25 m | 18(M:10,F:8)  Mean age: 40.5y  Mean disease  duration:  22m | 3m | N.R. | 1. Total clinical efficacy rate  2. EDSS  3. Average  length of Stay  4. Adverse events | 1. *P* < 0.05  2. *P* < 0.01  3. *P* < 0.05 |
| Zhou et al.2013(14) | McDonald | Shuganjianpigusui formula  1 dose/d(400 ml) + CT | MPPT1000 mg/d ivgtt  gradully halved every 3 d  to 120 mg/d + Prednisone  60 mg/d po  reduced weekly 10 mg/d | 14(NR)  Mean age:  48.86 ± 10.54y  Mean disease  duration:  43.36 ± 39.7 m | 21(NR)  Mean age:  46 ± 10.25y  Mean disease  duration:  48.38 ± 40.52 m | 3w | N.R. | 1. Annual relapse frequency  2. Annual relapse interval | 1. *P* < 0.05  2. *P* < 0.05 |
| Pu 2012(15) | McDonald | CHM 1 dose/d(200 ml) + CT | MPPT 1000 mg/d for  3d + Prednisone 60 mg/d | 22 (M:80,F:14)  Mean age:  34.5 ± 12.69y  Mean disease  duration: 16.3 m | 21(M:7,F:14)  Mean age:  38.95 ± 14.09y  Mean disease  duration: 17.1m | 1m | N.R. | 1. EDSS  2. Total clinical efficacy rate | 1. *P* < 0.05  2. *P* < 0.05 |
| Wei 2012(16) | McDonald | CHM 1 dose/d + CT | MPPT 1000 mg/d ivgtt  gradually halved every  3d to 120 mg/d + Prednisone  60 mg/d reduced weekly  10 mg/d po | 25(M:4,F:21)  Mean age: N.R  Mean disease  duration: N.R | 20(M:5,F:15)  Mean age: N.R  Mean disease  duration: N.R | 3m | N.R. | 1. Annual relapse interval | 1. *P* < 0.05 |
| Li and Zhao 2012(17) | McDonald | CHM decoction 1 dose/d + CT | MPPT 500–1000 mg/d + 5%Glu 500 ml ivgtt for 5d + Prednisone 60 mg/d po | 30(M:12,F:18)  Mean age:  40.24 ± 2.53y  Disease duration:  3.31 ± 1.25y | 30(M:13,F:17)  Mean age:  42.31 ± 1.24y  Disease duration:  3.26 ± 1.08y | 3m | N.R. | 1. EDSS  2. Total clinical efficacy rate | 1. *P* < 0.01  2. *P* < 0.05 |

**Table 1** (Continued)

| Study | Diagnosis criteria | Trial | Control | Trial  (male/female; age;Duration) | Control  (male/female; age;Duration) | Treatment during | Follow-up peried | Outcome measure | Intergroup Differences |
| --- | --- | --- | --- | --- | --- | --- | --- | --- | --- |
| Zeng et al.2009(18) | Poser | Buyanghuanwu decoction  plus-minus 1 dose/d + CT | MPPT 1000 mg/d + NS  500 ml ivgtt for 5d + Prednisone 60 mg/d | 35(M:15,F:20)  Mean age: 42y  Mean disease  duration: N.R | 30(M:11,F:19)  Mean age: 40y  Mean disease  duration: N.R | 1m | N.R. | 1. Total clinical efficacy rate  2. Adverse events | 1. *P* < 0.05 |
| Yang et al.2009(19) | McDonald | Simiaoyongan decoction and marrow storing pill plus-minus 1 does/d | MPPT 1000 mg/d + 5%Glu  500 ml ivgtt for 5d +Prednisone 60 mg/d po | 30(M:11,F:19)  Mean age:  35.67 ± 12.23y  Mean disease  duration: N.R | 15(M:5,F:10)  Mean age:  35.17 ± 12.64y  Mean disease  duration: N.R | 3m | N.R. | 1. Total clinical efficacy rate | 1. *P* < 0.05 |
| Gao et al.2008(20) | Poser | Dihuanheji Capslue 4#tid + CT | MPPT 500–1000 mg/d + 5%Glu 500 ml ivgtt for 5d + Prednisone 25–50 mg/d po | 38(M:21,F:17)  Mean age:  37.10 ± 7.56 y  Mean disease  duration: N.R | 40(M:24,F:16)  Mean age:  36.35 ± 7.67y  Mean disease  duration: N.R | 3w | 1y | 1. EDSS  2. Annual relapse frequency | 1. *P* < 0.001  2. *P* < 0.05 |
| Fan et al.2007(21) | Poser | Erhuang formula + CT | MPPT 1000 mg/d ivgtt for  6d + Prednisone 60 mg/d | 30(M:9,F:21)  Mean age:  38.1 ± 12.48 y  Mean disease  duration: N.R | 35(M:8,F:27)  Mean age:  36.46 ± 14.13y  Mean disease  duration: N.R | N.R. | 1y | 1. EDSS  2. Annual relapse frequency | 1. *P* < 0.05  2. *P* > 0.05 |
| Wang et al.2006(22) | Poser | Jiweiling decoction 1 does/d + CT | MPPT 500 mg/d ivgtt for  5d + Prednisone 80 mg/d  halved every 7d–10 mg/d  po | 36(M:20,F:16)  Mean age:  26.25 ± 6.70y  Mean disease  duration:  2.58 ± 0.34 m | 32(M:14,F:18)  Mean age:  27.65 ± 5.8y  Mean disease  duration:  2.64 ± 0.41 m | 2m | N.R. | 1. Total clinical efficacy rate  2. Adverse events | 1. *P* < 0.01 |

**Table 1** (Continued)

| Study | Diagnosis criteria | Trial | Control | Trial  (male/female; age;Duration) | Control  (male/female; age;Duration) | Treatment during | Follow-up peried | Outcome measure | Intergroup Differences |
| --- | --- | --- | --- | --- | --- | --- | --- | --- | --- |
| Zuo and Jia 2006(23) | Poser | Yishengujintongluo formula 1 dose/d + CT | MPPT 1000 mg/d + 5%Glu  500 ml ivgtt for 5d + Prednisone 40–60 mg/d reduce to 25–50 mg/d po | 30(M:13,F:17)  Mean age:  33.4 ± 10.5y  Mean disease  duration:  17.3 ± 11.8 m | 30(M:12,F:18)  Mean age:  31.9 ± 12.3y  Mean disease  duration:  16.8 ± 12.1 m | 2m | N.R. | 1. Total clinical efficacy rate  2. EDSS | 1. *P* > 0.05  2. *P* < 0.01 |
| Zhang and Zhang 2006(24) | McDonald | Gusuitongluo decoction  1 does/d + CT | MPPT 1000 mg/d + 5%Glu  500 ml ivgtt for 5d +Prednisone 40–60 mg/d  reduce to 25–50 mg/d po | 30(M:13,F:17)  Mean age:  33.41 ± 10.52y  Mean disease  duration:  17.32 ± 11.82 m | 30(M:12,F:18)  Mean age:  31.93 ± 12.31y  Mean disease  duration:  16.82 ± 12.14 m | 2m | N.R. | 1. EDSS  2. Total clinical efficacy rate | 1. *P* < 0.05  2. *P* < 0.05 |
| Shi and Wang 2004(25) | Poser | Jiannaogusui decoction 1  dose/d + CT | MPPT 1000 mg/d + 5%Glu  500 ml ivgtt for 5d + Prednisone 25–50 mg/d | 19(M:7,F:12)  Mean age:  32.5 ± 11.6y  Mean disease  duration:  40.5 ± 37.6 m | 19(M:8,F:11)  Mean age:  33.1 ± 10.2y  Mean disease  duration:  41.3 ± 31.53 m | 3m | 1.0–2.5 y | 1. Total clinical efficacy rate  2. EDSS  3. Annual relapse frequency  4. Adverse events | 1. *P* < 0.05  2. *P* < 0.01  3. *P* < 0.01 |

Note: CHM, Chinese herbal medicine; MPPT, methylprednisolone; N.R., non reported; EDSS,Expanded Disability Status Scale; CT, control therapy; d, day(s); w, week(s); m, month(s); y, year(s); tid, ter in die; bid, bis in die; ivgtt, intravenously guttae; po, per os; M, male; F, female

**References**

1. Wu P, Xie JE, Chen F, Song QY. Clinical Observation on Nourishing Liver and Kidney Recipe in the Treatment of Multiple Sclerosis. *Chinese Medicine Modern Distance Education of China* (2020) 18(11):84-6.

2. Shi LM. Clinical Observation of Impoved Pingfu Decoction in Adjuvant Treatment of Multiple Sclerosis. *Journal of Practical Traditional Chinese Medicine* (2020) 36(3):361-2.

3. Qian BC, Wang BL. Integrated Traditional Chinese and Western Medicine Treatment of 3 Cases of Acute Stage Multiple Sclerosis *Traditional Chinese Medicinal Research* (2020) 33(04):16-8.

4. Huang C, Zhou ZY, Liu GW. Twenty-One Cases of Multiple Sclerosis Treated with

Formula of Tonifying the Kidney and

Supplementing the Essence. *Henan Traditional Chinese Medicine* (2018) 38(09):1409-12. doi: 10.16367/j.issn.1003-5028.2018.09.0377.

5. Fan YP, Chen KL, You YZ, Wang S, Yang T, Wang JL. Clinical Efficacy Observation of Bushen Yisui Capsule on Relapsing Remitting Multiple

Sclerosis with Syndrome of Deficiency of Kidney-Liver Yin. *China Journal of Traditional Chinese Medicine and Pharmacy* (2018) 33(09):4220-3.

6. Li SH. Clinical Observation of Tripterygium Wilfordii Polyglycosides in the Treatment of Multiple Sclerosis. *Research of Integrated Traditional Chinese and Western Medicine* (2017) 9(06):288-9+92.

7. Wu P, Xie JE, Huang LW, Chen W, Liang H. Effect Observation on Multiple Sclerosis with Yanggan Zishen Decoction. *Journal of Shanxi University of Chinese Medicine* (2016) 17(4). Epub 20170128.

8. Lu LP. Clinical Observation on 35 Cases of Multiple Sclerosis Treated by Heat-Clearing and Detoxifying Method. *China Health Care & Nutrition* (2016) 26(11):78-9.

9. Chen KL, Fan YP. Effect of Kidney － Nourishing，Phlegm － Ｒesolving and Blood － Activating

Method on Quality of Life in Patients with Multiple Sclerosis. *Chinese Archives of Traditional Chinese Medicine* (2016) 34(09):2141-4. doi: 10.13193/j.issn.1673-7717.2016.09.026.

10. Chen L, Wang T. Clinical Observation of Dihuangheji in the Treatment of Elderly Patients with Multiple Sclerosis. *China Health Care & Nutrition* (2016) 26(30):240-. doi: 10.3969/j.issn.1004-7484.2016.30.382.

11. Li HH, Chen Q. Clinical Analysis of Integrated Traditional Chinese and Western Medicine Treatment of Multiple Sclerosis. *Contemporary Medicine* (2015) 21(18):158-9.

12. Zhou L, Fan YP. Randomized Trial of Erhuangfang for Relapsing Multiple Sclerosis. *Neurological Research* (2015) 37(7):633-7. doi: <https://dx.doi.org/10.1179/1743132815Y.0000000011>.

13. Zhao WN. Integrated Traditional Chinese and Western Medicine Treatment of 36 Cases of Multiple Sclerosis *China Journal of Pharmaceutical Economics* (2013) (01):55-6.

14. Zhou YQ, Mao WQ, Zhang XJ, Li T. Effects of Shugan Jianpi Gusui Recipe on Multiple Sclerosis Recurrence: A Primary Report. *Chinese Journal of Integrated Traditional and Western Medicine* (2013) 33(01):31-4.

15. Pu GF. Clinical Effect Observation of Kidney-Based Syndrome Differentiation and Treatment of Multiple Sclerosis: Beijing university of traditional chinese medicine (2012).

16. Wei Q. Clinical Study on Treatment of Multiple Sclerosis with Modified Sini Powder [硕士]: Beijing university of traditional chinese medicine (2012).

17. Li Q, Zhao D. Integrated Traditional Chinese and Western Medicine Treatment of 30 Cases of Multiple Sclerosis *Traditional Chinese Medicinal Research* (2012) 25(01):22-3.

18. Zeng HM, Zhang M, Zhang G, Zi XF. A Clinical Effect Observation on the Buyang Huanwu

Decoction with Hormone Treatment for Multiple Sclerosis. *Practical Clinical Medicine* (2009) 10(10):9-10+4.

19. Yang HF, Lv EB, Sun CQ. Simiao Yong 'an Decoction Combined with Fengsui Dan in the Treatment of Multiple Sclerosis. *Chinese Medicine Modern Distance Education of China* (2009) 7(09):21-2.

20. Gao M, Lin MC, Zhang KN, Lin YY, Lv N. Clinical Observation on 38 Cases of Acute Recurrent Multiple Sclerosis Treated with Dihuangheji ( Capsule ). *Hunan Journal of Traditional Chinese Medicine* (2008) (06):16-7. doi: 10.16808/j.cnki.issn1003-7705.2008.06.008.

21. Fan YP, Wang P, Zhang XH, Gong HY, Zhou L, Liu XZ, et al. Mechanism Exploration of Erhuang Formula in Treating Acute Episode

of Disseminated Sclerosis. *China Journal of Traditional Chinese Medicine and Pharmacy* (2007) (01):25-9.

22. Wang YH, Zhao H, Huang JH, Su ZL. Clinical Study of Jiweiling Decoction in the Treatment of Multiple Sclerosis. *Modern Journal of Integrated Traditional Chinese and Western Medicine* (2006) (12):1608-9.

23. Zuo SZ, Jia YL. Yishen Gujin Tongluo Decoction Plus Western Medicine in the Treatment of 30 Cases of Multiple Sclerosis. *Traditional Chinese Medicinal Research* (2006) 19(8):30-2. doi: 10.3969/j.issn.1001-6910.2006.08.016.

24. Zhang GZ, Zhang JS. Clinical Study of Gusui Tongluo Decoction in the Treatment of Multiple Sclerosis. *Journal of Emergency in Traditional Chinese Medicine* (2006) (06):595-6.

25. Shi LH, Wang QW. Preliminary Study on the Effect of Integrated Traditional Chinese and Western Medicine on the Prevention and Treatment of Recurrence of Multiple Sclerosis. *Guangxi Journal of Traditional Chinese Medicine* (2004) (02):14-7.
